# Evidence Communication Rules for Policy (ECR-P) critical appraisal tool

**DOI:** 10.1186/s13643-025-02757-8

**Published:** 2025-01-13

**Authors:** Evangelos Danopoulos, John A. D. Aston, Aarushi Shah, Claudia R. Schneider

**Affiliations:** 1 Statistical Laboratory, Department of Pure Mathematics and Mathematical Statistics, University of Cambridge, Cambridge, UK; 2 School of Psychology, Speech and Hearing, University of Canterbury, Christchurch, New Zealand

**Keywords:** Critical appraisal, Quality assessment, Risk of bias tool, Policy recommendations, Policymaking

## Abstract

**Background:**

Scientific papers increasingly put forward scientific-based policy recommendations (SPRs) as a means of closing the circle of science, policy and practice. Assessing the quality of such SPRs is crucial, especially within the context of a systematic review. Here, we present ECR-P (Evidence Communication Rules for Policy)—a critical appraisal tool that we have developed, which can be used in assessing not only the quality of SPRs but also the quality of their evidence base and how effectively these have both been communicated.

**Methods:**

The rationale behind ECR-P centres on three dimensions of quality; two are the well-established concepts of *internal* and *external validity*. Here, we introduce a third—*evidence communication*—encompassing both evidence veracity and quality of communication. Elements of the three dimensions of quality are considered within the context of the five rules of evidence communication. These are as follows: inform, not persuade; offer balance, not false balance; disclose uncertainties; state evidence quality and pre-empt misunderstandings.

**Results:**

Development of ECR-P has been carried out by an interdisciplinary team and was piloted with a systematic review reported more fully elsewhere. ECR-P comprises a set of preliminary considerations which capture key aspects for the assessment, leading on to the main tool whose structure is domain-based, each domain mapping to one of the five rules of evidence communication. The domains include 25 signalling questions designed to obtain essential information for the critical appraisal. The questions focus on either the study’s evidence or the policy recommendations. Domain-based judgement is derived from responses to the signalling questions and an accompanying algorithm, followed by an overall quality judgement.

**Conclusions:**

ECR-P has been designed to provide a standardised and transparent approach to assess the quality and communication of SPRs and their evidence base. The tool, which could be applied across all scientific fields, has been developed to fit primarily with the systematic reviewing process but could also serve as a stand-alone tool. Besides review assessors, it can also be used by policymakers, researchers, peer reviewers, editors and any other stakeholders interested in evidence-based policymaking and high-quality evidence communication. We encourage further independent testing of the tool in real-world evidence-based research.

**Supplementary Information:**

The online version contains supplementary material available at 10.1186/s13643-025-02757-8.

## Background

There is widespread consensus that science and evidence should underpin policy [1]. Indeed, scientific papers increasingly present policy implications of their findings and/or put forward scientific-based policy recommendations (SPRs) thereby influencing the policymaking process. It is often recognised, however, that significant barriers still exist in knowledge exchange between scientists and policymakers [2].

The use of evidence-based policies was first established in health care and is now rapidly spreading into other areas of public life [3]. The overarching benefit of evidence-based policymaking is that it relies heavily on the use of scientific evidence and avoids political motivation. Informing future policy based on data that have been identified, collected and synthesised in a transparent and reproducible manner is the cornerstone of evidence-based policymaking [4]. This is where systematic reviews come into play. Systematic reviews should also incorporate a critical appraisal of the quality of each individual study, also often termed risk of bias (RoB) assessment [5]. The critical appraisal outcomes can be used to identify the best available evidence and to appraise the certainty of the body of evidence [6].

There are several existing critical appraisal tools focusing on different areas of research. The majority of these tools have been developed for medical research (e.g. RoB2 [7], ROBINS-I [8], the Newcastle–Ottawa Scale [9]), while there are also tools developed specifically for environmental studies [6, 10, 11]. These tools focus on the scientific outcomes of the papers. As more policy recommendations are reported in scientific papers and are likely to be used to underpin evidence-based policymaking, a tool facilitating their quality assessment is needed. A method of peer evaluation of these aspects is as critical to a systematic review process as any other aspect of the paper.

Here, we present ECR-P (Evidence Communication Rules for Policy), a critical appraisal tool which can be used to assess the quality and communication of a study’s evidence base and the quality and communication of SPRs. Our goal here is to report our critical appraisal tool, outlining how it was created and, with the help of the accompanying explanation and elaboration document (Additional file 1), to provide sufficient information on how to use it.

## Methods

### Definitions and scope

#### Dimensions of quality

**Fig. 1:**
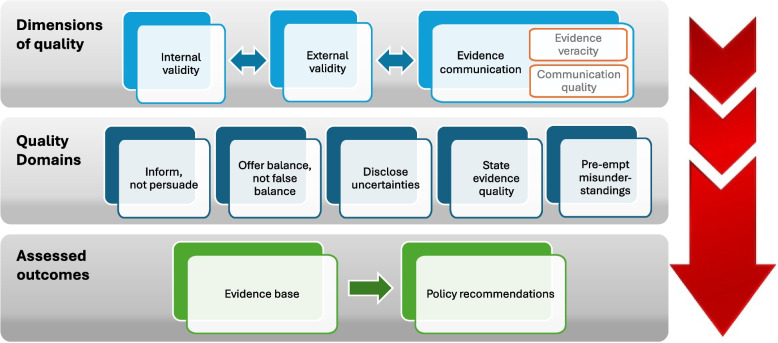
ECR-P (Evidence Communication Rules for Policy) critical appraisal tool conceptual framework. ECR-P critical appraisal covers elements of three interconnected dimensions of quality that are examined within the context of five quality domains. The quality domains correspond to the five rules for evidence communication [12]. The scope of the assessment are the study’s evidence base (findings and conclusions) and the policy recommendations

The ECR-P critical appraisal tool comprises three interconnected dimensions of quality: *internal validity*, *external validity* and *evidence communication* (see Fig. 1). *Internal validity* refers to the extent that systematic error or bias (deviation from the truth [3]) has been introduced in the outcomes of the study. Potential sources of bias are flaws in the study’s design, conduct, analysis or reporting [4]. *External validity* refers to generalizability, i.e. the transportability and applicability of study outcomes for the objectives of the review [3].

*Evidence communication* is introduced as a new dimension of quality that complements the established dimensions of internal and external validity. Assessment of evidence communication is based on two pillars: evidence veracity and quality of communication [13]. Evidence veracity refers to factual accuracy of the communication. Quality of communication refers to the extent to which the study’s reporting style and language adheres to specific communication principles, as discussed further below. Therefore, the *modus* of communication is assessed in conjunction with the accuracy of the communication [14]. Both components are equally important. An inaccurate message that is communicated excellently has little value and can lead to negative repercussions, as illustrated by the rise of fake news [15]. On the other hand, an accurate message that has not been communicated effectively may not have the desired penetration or impact [16]. Both conditions must be met to achieve high-quality evidence communication.

The significance of these interconnected areas of evidence communication is illustrated in the established guidance for developing trustworthy health care guidelines [17]. This comprehensive guidance includes several topics on how evidence and their quality should be assessed, used and accurately communicated. Furthermore, the guidance provides advice on specifics of communication, such as wording, reporting and dissemination, highlighting the relationship between these areas [18].

#### Five rules for evidence communication

The concept behind ECR-P was informed by *the five rules for evidence communication*: inform, not persuade; offer balance, not false balance; disclose uncertainties; state evidence quality; inoculate against misinformation [12]. Through ECR-P, we intended to *operationalise* these principles for evidence communication excellence into a critical appraisal tool grounded in the three dimensions of quality (Fig. 1).

The five rules for evidence communication were developed by the Winton Centre for Risk and Evidence Communication [19] who worked extensively on the interface between evidence communication and decision making. The aim of the principles is to guide the design of trustworthy and high-quality evidence communication that facilitates evidence-based decision making. The results of a recent empirical study support their use in communicating trustworthy messages to the public in order to aid decision making [13].

#### Outcomes assessed by ECR-P

The focus of the ECR-P critical appraisal tool is on two sets of study outcomes (Fig. 1). The first is the evidence base, reported in a study in the form of findings and conclusions. The second is the set of SPRs that stem from the evidence base. SPRs are defined as policy recommendations that are put forward in the context of any type of scientific study and should be guided and based on the scientific outcomes of the study. This connection is examined within ECR-P, since this is the essence of an SPR [16].

The goal was to develop a useful tool that would examine key elements of the three dimensions of quality within the context of the five rules of evidence communication focusing on both the evidence base and the SPRs, thus providing a holistic assessment of both quality and communication.

The methodological conduct of a study is not examined by ECR-P. Other, discipline-specific, RoB tools that have been specifically designed for this purpose should be used accordingly in the reviewing process.

### Development of the critical appraisal tool

In developing ERC-P, we drew on a project team with diverse backgrounds and expertise ranging from mathematics and policymaking to evidence-based research, evidence communication, medical science, research methodology and psychology. Other researchers, academics and stakeholders were also asked for feedback during the development of the tool, mainly drawing from a project focusing on real-time digital optimisation and decision making for energy and transport systems. This ongoing project involves four universities, policymakers and industry partners [20].

The development of ECR-P was guided by a published framework for developing quality assessment tools [21] and established methods of evidence-based research [4, 5, 22]. As such, we aimed to develop a domain-based tool, comprised of signalling questions that prompt critical appraisal judgements on each domain and overall, similar to existing critical appraisal tools [7, 8].

The piloting and validation of the tool was based on the execution of a systematic review focusing on SPRs for tackling climate change and reaching net zero target via the use of green energy [23]. Many iterations of the tool were trialled before the final version was agreed. Piloting was executed independently by all co-authors and inter-rater agreement was used as a validation metric. During this phase, the signalling questions were refined and their respective weights for reaching a judgement were agreed. The final version of the tool was decided by consensus between all co-authors. The overall choice of the quality domains and signalling questions was based on the empirical evidence examined in the systematic review, previous experience in policymaking environments and theoretical reflections.

## Results

### Preliminary considerations

**Fig. 2:**
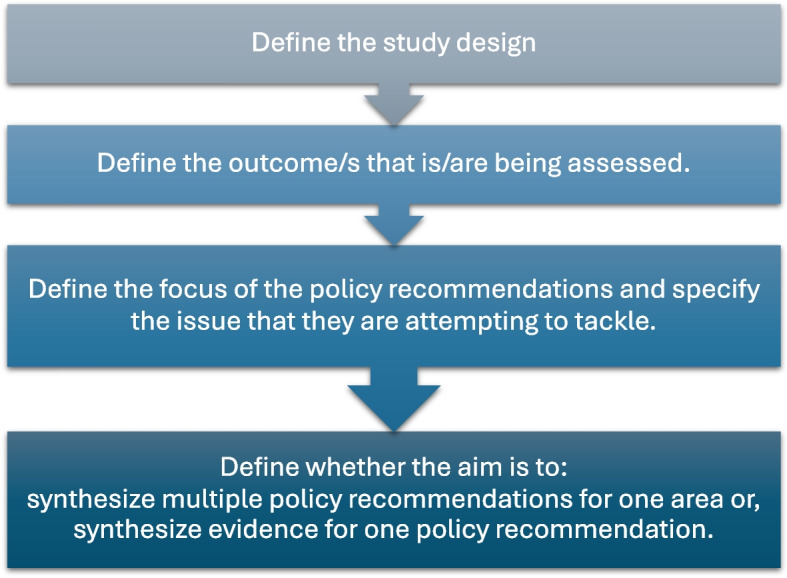
ECR-P (Evidence Communication Rules for Policy) critical appraisal tool preliminary considerations

The process of the critical appraisal is comprised of two phases: the preliminary considerations and the main critical appraisal tool. Before embarking on the critical appraisal assessment, it is useful for the assessors to identify important aspects of the study which will guide the appraisal process (Fig. 2). The study design and the research outcomes are key characteristics and should be captured. Moreover, the assessors should examine the SPRs put forward by the study and define whether they will focus on all of the recommendations or only on specific ones, as guided by the review question/s.

A review (systematic or not) of policy recommendations might aim to identify and synthesize various policy recommendations relevant to one area or issue or multiple areas and issues. On the other hand, a review might aim to focus on a specific policy recommendation made for a specific issue, bringing together evidence from various sources. If only part and not all of the policy recommendations are included in the scope of the review question, it should be defined early on in this preliminary stage as it will guide the appraisal process.

### ECR-P domains

ECR-P consists of five domains, each corresponding to one of the five rules of evidence communication [12]. The overview of the tool is presented in Table 1. Each domain includes a set of signalling questions that are further grouped into two levels: the study level and the policy recommendations level. The first focuses on the evidence base (findings and conclusions) of the study, and the second focuses on the SPRs of the study.

**Table 1:** Evidence Communication Rules for Policy (ECR-P) critical appraisal tool

Domain and signalling question	Response options and judgement		
	Lower RoB	Higher RoB	Other
**Domain 1: Inform not persuade**			
***Study level***			
1.1 Were the aims/objectives for the study defined?	Y/PY	N/PN	NI
1.2 Were the limitations of the study findings reported?	Y/PY	N/PN	NI
If Y/PY to 1.2: 1.2.1 Did the study propose ways to reduce limitations in the future?	Y/PY	N/PN	NI/NA
1.3 Were the study conclusions clearly connected to the findings of the study?	Y/PY	N/PN	NI
1.4 Was emotive language avoided in communicating study findings and/or conclusions?	Y/PY	N/PN	NI
***Policy recommendations level***			
1.5 Were the aims/objectives for the policy recommendations defined?	Y/PY	N/PN	NI
1.6 Were the limitations of the policy recommendations reported?	Y/PY	N/PN	NI
1.7 Were the policy recommendations clearly connected to the findings of the study?	Y/PY	N/PN	NI
1.8 Was accessible language used for the policy recommendations?	Y/PY	N/PN	NI
1.9 Was emotive language avoided in policy recommendations?	Y/PY	N/PN	NI
**Domain 2: Offer balance, not false balance**			
***Study level***			
2.1 Were all aspects of the study findings reported?	Y/PY	N/PN	NI
2.2 Was an appropriate reporting guideline used for constructing the manuscript?	Y/PY	N/PN	NI
***Policy recommendations level***			
2.3 Were multiple implications of the policy recommendations considered?	Y/PY	N/PN	NI
2.4 Was the existence of a current policy discussed?	Y/PY	N/PN	NI
If Y/PY to 2.4 2.4.1 Was not changing the current policy considered?	Y/PY	N/PN	NI/NA
**Domain 3: Disclose uncertainties**			
***Study level***			
3.1. Were uncertainties of the study findings reported?	Y/PY	N/PN	NI
If Y/PY to 3.1 3.1.1 Did the study propose ways to reduce uncertainties in the future?	Y/PY	N/PN	NI/NA
***Policy recommendations level***			
3.2 Were uncertainties of the policy recommendations reported?	Y/PY	N/PN	NI
If Y/PY to 3.2 3.2.1 Did the study adopt a precautionary principle perspective?	Y/PY	N/PN	NI/NA
**Domain 4: State evidence quality**			
***Study level***			
4.1 Was the quality of the evidence used in the analysis considered**?	Y/PY	N/PN	NI
If Y/PY to 4.1 4.1.1 Were specific metrics of evidence quality used?	Y/PY	N/PN	NI/NA
***Policy recommendations level***			
4.2 Was the quality of the study findings, that formulated the evidence base for the policy recommendations, considered?	Y/PY	N/PN	NI
**Domain 5: Pre-empty misunderstandings**			
***Study level***			
5.1 Were potential misunderstandings about the study findings and conclusions pre-emptively addressed?	Y/PY	N/PN	NI
***Policy recommendations level***			
5.2 Was the targeted audience for policy recommendations defined?	Y/PY	N/PN	NI
5.3 Were potential misunderstandings for policy recommendations and potential concerns of the policy makers pre-emptively addressed?	Y/PY	N/PN	NI

*N *no, *NA *not applicable, *NI *no information, *PN *probably no, *PY *probably yes, *RoB *Risk of Bias, *Y *yes

#### Inform, not persuade

This domain aims to assess whether researchers have been open about motivations and limitations. It has been established that trustworthiness of communication is judged not only by expertise and honesty but also by evidencing good intentions [24]. Authors should be clear about the aims and objectives of their study and disclose any factors that introduce limitations to their findings and the consequent policy recommendations. Ideally, recognising limitations should also be accompanied by proposing future solutions to mitigate them. A clear connection between the study findings and conclusions and policy recommendations is key in evidencing trustworthiness of communication.

In communicating evidence, authors should present findings and policy recommendations in a neutral manner. Emotive language, which can be persuasive, should be avoided. Emotive language is wording that is used in order to elicit an undue emotional response in the reader. Undue, here, refers to trying to evoke an emotion based on unsubstantiated statements. In addition, when authors are composing the policy recommendation section, they should keep in mind that their readers will include policymakers who might not be familiar with their scientific field. Therefore, they should strive to avoid scientific jargon as much as possible in the policy recommendations section to improve both accessibility and informativeness.

#### Offer balance, not false balance

In this domain, the balance of the communicated evidence is assessed. In the interest of informing fully, a balanced account of evidence should be provided. The focus is first on the completeness of reporting. All aspects of study results should be reported whether a hypothesis has been verified or not. In many scientific fields, a guideline is followed in order to exhibit the quality and completeness of reporting (e.g. CONSORT [25], STROBE [26], CHEERS [27] etc.). Implementing such a guideline is advisable. Regarding the policy recommendations section, all foreseeable aspects of a recommended policy should be discussed, whether positive or negative. Knowledge of the policy status quo and how this might be affected should also be demonstrated. Developing and implementing a new policy is very likely to have negative implications as well. Authors should investigate and report on their SPRs’ implications always keeping in mind the end user. End users in this sense being both those who will enforce the policy recommendations as well as the groups affected by them.

#### Disclose uncertainties

This domain focuses on whether *what we don’t know* is clearly communicated. A strategy that could be adopted is for authors to state: what they know; what they don’t know; what research could be done in the future to find out more; what people could do in the meantime to be on the safe side; and that initial recommendations might be subject to change [12, 28]. Reporting uncertainties might be seen to be easier and more straightforward if studies included quantitative analysis (e.g. statistical confidence intervals). Nevertheless, studies should also explain contextually what this uncertainty means in terms of their findings. In addition, authors must consider how the uncertainty of their findings might create uncertainty in their policy recommendations. If uncertainty exists, it could be the case that the precautionary principle should be adopted in the meantime [29].

#### State evidence quality

This domain addresses the communication of evidence quality. The credibility of a communication and the reliability of a study finding can be affected by the quality of the underlying evidence. Whether the data used in a study is primary, collected by the authors, or secondary, retrieved from other sources, its quality should be reported and considered. The use of a standardised quality metric, if one exists for the specific area, is advisable. Undoubtedly, the quality of the underlying scientific evidence that the study is based upon will affect the quality of the policy recommendations that have been developed based on them. A consideration of this interaction is key.

#### Pre-empt misunderstandings

This domain addresses the repercussions that can stem from inadequate understanding, be it due to a lack of adequate information, a lack of clarity in presented information, an overload of information that reduces clarity, for instance, for non-experts, or the existence of misinformation, to name but a few potential scenarios. Effort must be made to pre-empt misunderstandings and inoculate against misinformation via “*prebunking*” [30]. In order to achieve this goal, one must anticipate potential issues arising from misunderstandings or even disinformation. Especially in the policy recommendations section, knowing your audience and offering clear and practical recommendations can help address this issue.

### Signalling questions

Signalling questions were designed to obtain information around one or more dimensions of quality addressing the context of each rule for evidence communication. It should be noted that there is significant interplay and some overlap between certain elements of the dimensions of quality that are examined by ECR-P. Mapping to the three dimensions of quality is presented in Additional file 2.

The available responses for each signalling question are predefined (Table 1). The affirmative responses ‘Yes’ and ‘Probably Yes’ are associated with low concerns for RoB and therefore high-quality outcomes. Consequently, the negative responses ‘No’ and ‘Probably No’ are associated with high concerns for RoB and low-quality outcomes. The options ‘Probably Yes’ and ‘Probably No’ should be used in the case where the determination had to fall back on a judgement made by the assessor. Such judgements are made when clear objective evidence is not available in the study but can be safely inferred from the context. The ‘No information’ response should be used when not enough information is reported for the assessor to make a ‘probably yes’ or ‘probably no’ judgement within the context of the study. The ‘not applicable’ option is mainly used when a signalling question is connected to a previous one that has not been answered positively. Nuances of the different responses for each of the signalling questions are described in detail in the elaboration and explanation document (see Additional file 1). Each response must be justified in a free text box. Assessors may use direct quotations from the papers to justify their responses when possible.

### Domain and overall judgement

The critical appraisal assessment results are expressed as RoB judgements. The term RoB is often used to describe concerns regarding the results of a study specifically arising from areas of internal validity [4]. We decided to use the same term here for two reasons. First, ECR-P touches upon key internal validity issues, especially in the study level of each domain (see Additional file 2). Second, assessors are familiar with this term being used in the quality appraisal process within systematic reviews, as established by many existing quality appraisal tools. Third, using this terminology would facilitate a seamless incorporation of ECR-P assessment outcomes into frameworks for developing body of evidence summaries such as GRADE (Grading of Recommendations, Assessment, Development, and Evaluations) [31].

RoB judgement will be reached for each domain separately and there will also be an overall RoB judgement. RoB judgement per level of domain will be based on an algorithm depending on the responses to each of the signalling questions. Consequently, the ratings of the two levels will be combined in a rating per domain. The algorithms are provided in the accompanying explanation and elaboration document (see Additional file 1). It should be noted that the decision trees are our suggestion so the judgement can be overridden by the assessors by providing appropriate justifications. The algorithm incorporates considerations of both the study level as well as the policy recommendations level within each domain.

The RoB judgement options for domain and overall judgement are as follows: Low risk of bias; Some concerns and High risk of bias, corresponding to high, moderate and low quality. The overall RoB judgement is mapped to the domain level judgements. The worst rating across domains will be carried over to the overall RoB judgement. We advise that a paper should be rated as low RoB, indicating the highest quality, only when all domains have low RoB.

### Target users

ECR-P was initially designed to fit into the systematic reviewing process but also as a tool to be used in evidence-based policymaking. ECR-P can be used more generally to critically appraise SPRs in any domain. Potential users of the tool include policymakers and policymaking organisations of all levels as well as researchers and funders that are interested in evidence-based policymaking. Furthermore, ECR-P can be a useful tool in the peer-review process providing a clear and transparent critical appraisal roadmap for manuscript reviewers and journal editors. Target users can be expanded in the future with user-defined refinement.

## Discussion

ECR-P is the first tool, to our knowledge, designed to critically appraise the quality and the communication of SPRs and their evidence base, in any scientific discipline or area of policymaking. Evidence-based policymaking was always at the forefront of our thinking during the development of the tool. ECR-P was created with the intention to be used in the context of a systematic review. During the development of the tool, it became clear that it can also be helpful when used outside the narrow context of a review.

Quality appraisal of studies’ outcomes is an integral part of review processes. It is not a box-ticking exercise. The results of the assessment are to be used in the synthesis of the evidence, whether narrative or meta-analysis. In addition, ECR-P assessment results can be used within a framework for assessing the certainty of a body of evidence, for instance, within a systematic review such as the GRADE approach [4, 31].

Furthermore, the tool can be used to appraise the quality of a limited number of studies within the context of a rapid review. Rapid reviews may be executed in time-sensitive situations, where reliable evidence must be gathered quickly. On the other hand, the tool may also be used to examine a single study that is considered in a less formal context, for example, to inform a policy briefing.

ECR-P has a domain-based structure guided by the five rules for evidence communication. It was piloted and validated in a recent systematic review [23]. The structure and the operation of the tool is similar to existing and validated critical appraisal tools such as RoB2 for randomised controlled studies [7] and PROBAST for prediction model studies [32]. Each domain has a set of signalling questions guiding the identification and retrieval of vital information for quality appraisal. Both the study findings/conclusions and the policy recommendations as well as their communication, within the study context, are assessed.

Policy recommendations, in particular, present an additional risk regarding potential conflicts of interest, whether financial or otherwise. We decided not to include such considerations in the present tool. These issues should be addressed within the context of any (systematic) review. Specific frameworks for accessing conflict issues have recently started to be developed [33].

An increasing number of studies are putting forward SPRs. This practice is recognised as a positive step towards closing the gap between science and policymaking. Nevertheless, it can only be useful if the policy recommendations are grounded in scientific evidence and are of the highest quality.

We anticipate a growing number of (systematic) reviews of policy recommendations in various fields. ECR-P is particularly flexible. It can be used for quality assessment in the synthesis of multiple policy recommendations around one area, coming from one or multiple studies. Conversely, it can be applied to appraise the evidence quality concerning one specific policy recommendation put forward by one or multiple studies. The explanation and elaboration document provides further details for implementing ECR-P as well as suggested algorithms for reaching an overall quality assessment (see Additional file 1).

In our recent systematic review, we identified that the quality of policy recommendations and the quality of their communication was lower compared to their scientific findings and conclusions [23]. Targeted efforts must be made to correct this imbalance. Policymakers should be using the latest, most relevant and most reliable evidence to guide their decisions. This can be a daunting task given the large amount of research being produced as well as the spread and complexity of disciplines that might be involved even in one piece of policy. ECR-P can facilitate transparent evidence-based policymaking.

We anticipate that the validity of the tool will be independently tested when it is applied and evaluated in practice in future evidence-based research [34]. Furthermore, as with any other critical appraisal method, we expect that ECR-P will evolve as methods and practical experience evolve [7, 35].

## Conclusions

ECR-P is a critical appraisal tool that can be used to appraise the quality and the communication of SPRs and their evidence base. The tool can be used within the context of a systematic review or as a stand-alone checklist to aid policymakers. The scope of ECR-P goes beyond the traditional span of critical appraisal tools, by incorporating consideration of the quality of evidence communication. We believe that it offers a comprehensive approach to critical appraisal with a clear focus on policy recommendations and the scientific evidence behind them. We anticipate that the adoption of the tool by a diverse array of potential users will benefit future SPRs.

While ECR-P has been tested and validated in the environmental science sphere, it is still to be seen how well it will translate into other areas of scientific study and policymaking. Additional empirical data, collected from a range of scientific disciplines, are needed for the further validation and the future evolution of the tool. We want to invite evidence-based researchers and other interested parties to implement and further test and validate aspects of the tool relating to rater reliability, accuracy of assessments and ease of use. We welcome feedback for optimisation.

## Supplementary Information

Additional file 1. ECR-P (Evidence Communication Rules for Policy) critical appraisal tool. Explanation and elaboration.

Additional file 2. ECR-P (Evidence Communication Rules for Policy) critical appraisal tool. Mapping of the ECR-P (Evidence Communication Rules for Policy) critical appraisal tool signalling questions to the three dimensions of quality: internal validity, external validity, evidence communication. Mapping to evidence communication is further specified for either element of evidence veracity or communication quality.

## Data Availability

Not applicable.

## Abbreviations

ECR-P: Evidence Communication Rules for Policy
GRADE: Grading of Recommendations, Assessment, Development, and Evaluations
RoB: Risk of bias
SPR: Scientific-based policy recommendation
