# Supplementary material for: Evidence Communication Rules for Policy (ECR-P) critical appraisal tool
Source: Syst Rev. 2025 Jan 13;14:10. doi: 10.1186/s13643-025-02757-8 (PMC11727712; doi:10.1186/s13643-025-02757-8)
Supplement: Supplementary file 1 — Additional file 1. ECR-P (Evidence Communication Rules for Policy) critical appraisal tool. Explanation and elaboration. [file 13643_2025_2757_MOESM1_ESM.pdf]

ECR-P (Evidence Communication Rules for Policy) critical appraisal tool

Additional file 1: Explanation and elaboration

Domain and overall rating

Risk of bias (RoB) rating is reached in a stepwise approach. First, a rating is given to each of the two levels: study level and policy recommendations level, within each domain separately. These two ratings are then combined to provide the domain rating. Finally, the ratings of all the domains are combined for the overall rating of a study. An algorithm is provided for each level in each domain. Algorithms are also provided for reaching a rating per domain combining both levels, taking into consideration the structure of each domain. Regarding the overall rating, the worst rating in any individual domain should be carried over. If even one of the domains has been rated to raise ‘some concerns’ of RoB, the overall rating should be ‘some concerns’. Likewise, if even one domain is rated as of ‘high RoB’, then the overall rating should be ‘high RoB’. A study should be rated as of ‘low RoB’ only when that is the case for each of the domains.

Domain 1: Inform not persuade

Study level

Signalling question	Elaboration	Responses
1.1 Were the aims/objectives for the study defined?	Transparency of the intentions of the researchers speaks to their trustworthiness. Being open and precise about intentions is key to guarantee unwavering focus on impartiality.<br>Answer “Y” if the aims and/or objectives of the study are stated in the introduction section of the paper. Answer “PY” if the authors state how their study contributes to the current knowledge in the introduction section of the paper.	Y/PY/N/PN/NI
1.2 Were the limitations of the study findings reported?	Limitations of the findings are connected to the methodology limitations, data input limitations and analysis limitations. These finally affect and ‘limit’ the study’s findings. Openness regarding the limitations of the findings speaks to the trustworthiness of the study.<br>Answer “Y” if the study acknowledges specific limitations of the findings. Answer ‘PY’ if limitations are acknowledged but not clearly identified.	Y/PY/N/PN/NI
If Y/PY to 1.2:<br>1.2.1 Did the study propose ways to reduce limitations in the future?	The next step after acknowledging existing limitations is proposing possible solutions in future research.<br>Answer ‘Y/PY’ if the study proposes specific examples of how existing limitations might be tacked by future research.	Y/PY/N/PN/NI/NA
1.3 Were the study conclusions clearly connected to the findings of the study?	The key issue here is whether a ‘clear’ connection between findings and conclusions can be identified. A summary of findings in the conclusions section and a clear connection to the conclusions would support a ‘Y/PY’ answer.<br>As specified in the ‘preliminary considerations’ section the focus of the assessor might not be the entirety of the outcomes but on specific ones. The judgement here should be based on the outcomes included in the systematic review, aligned to the review question/s and specified in the preliminary considerations.	Y/PY/N/PN/NI
1.4 Was emotive language avoided in communicating	Emotive language is the wording that is used in order to elicit an undue emotional response in the reader. Undue, refers to trying to evoke an emotion based on unsubstantiated statements. Emotive language can be used to	Y/PY/N/PN/NI

study findings and/or conclusions?	persuade readers which goes directly against the 'inform not persuade' rule for evidence communication. Depending on the context emotive language can be defined as statements that are absolute, definitive, or even coercive.<br>For example, when an absolute/definite statement is made which is unsubstantiated and not backed up by scientific evidence, it would be identified as emotive.<br>Answer "Y/PY" if no form of emotive language was used.	

Suggested algorithm for rating judgment of study level in domain 1:

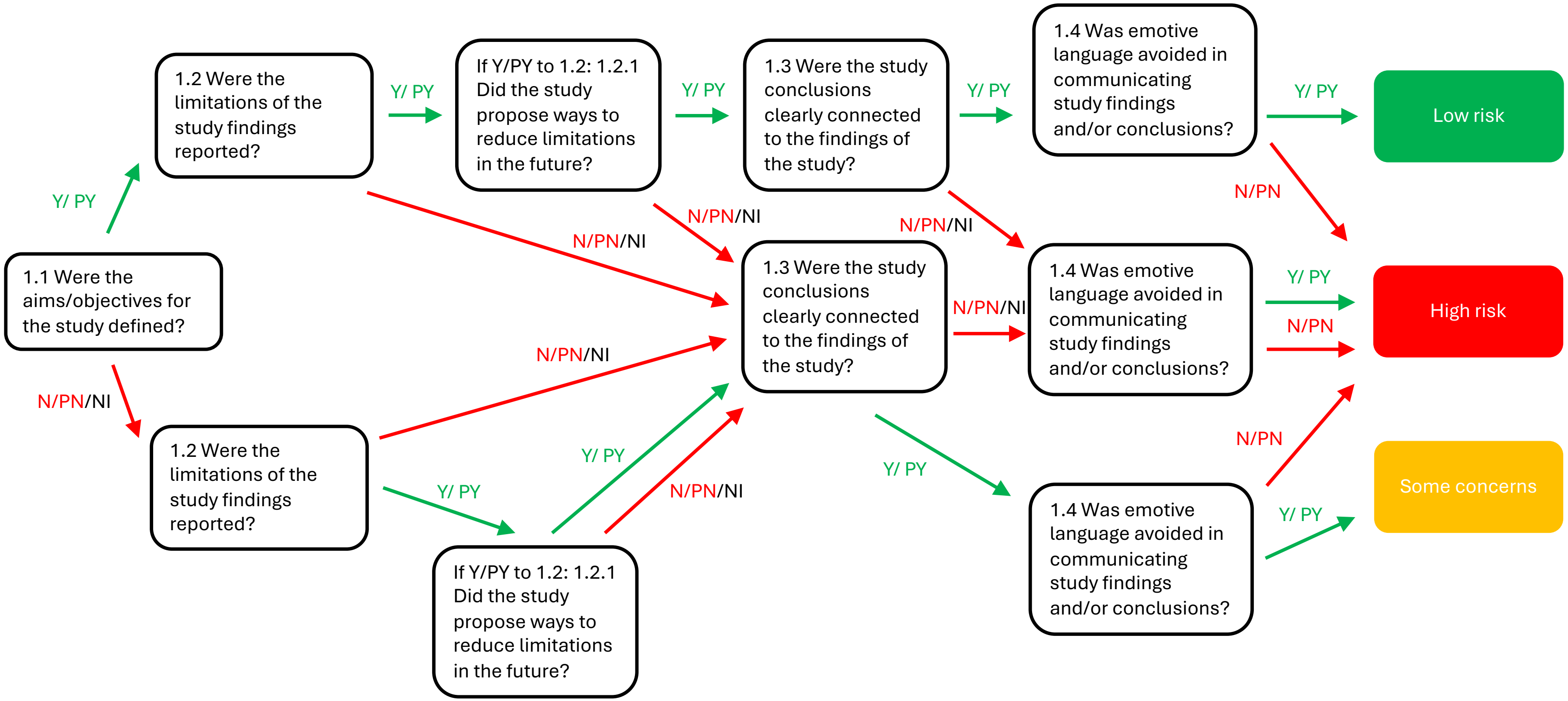

N, no; NA, not applicable; NI, no information; PN, probably no; PY, probably yes; Y, yes

Policy recommendations level

Signalling question	Elaboration	Responses
1.5 Were the aims/objectives for the policy recommendations defined?	Similarly to signalling question 1.1, researchers should state what their aims and/or objectives for the policy recommendations are. Answer ‘Y’ if the aims and/ or objectives of the policy recommendations are clearly stated in the paper. Answer ‘PY’ if the usefulness of the recommendations in a specific area is discussed.	Y/PY/N/PN/NI
1.6 Were the limitations of the policy recommendations reported?	Similarly to signalling question 1.2, being open about the limitations of the policy recommendations speaks to the trustworthiness of the study.<br>Answer “Y” if the study acknowledges specific limitations of the policy recommendations.<br>Answer ‘PY’ if limitations are acknowledged but not clearly identified.	Y/PY/N/PN/NI
1.7 Were the policy recommendations clearly connected to the findings of the study?	The policy recommendations must directly stem from the study findings. This is the essence of an evidence-based policy recommendation. A mere statement such as “according to our findings … these are the policy recommendations” is not enough. Answer ‘Y/PY’ if the connection between the study findings and the policy recommendations is clearly stated and justified.<br>If any unsubstantiated policy recommendations are put forward answer ‘N/PN’.<br>Similarly to question 1.3, the focus of the assessor might not be the entirety of the policy recommendations but on specific ones. The judgement here should be based on the outcomes included in the systematic review, aligned to the review question/s and specified in the preliminary considerations.	Y/PY/N/PN/NI
1.8 Was accessible language used for the policy recommendations?	Policy recommendations should not include scientific jargon. The wording of the policy recommendations should be accessible by non-expert policy makers.<br>Answer ‘Y/PY’ if scientific jargon was avoided and policy recommendations are written in plain language.	Y/PY/N/PN/NI
1.9 Was emotive language avoided in policy recommendations?	Similarly to signalling question 1.4, emotive language (absolute, definitive, coercive) should be avoided in the policy recommendations as well. Avoiding the use of emotive language in policy recommendations could be more problematic as researchers might be tempted to use ‘stronger’ language in order to persuade potential policy makers. Answer “Y/PY” if no form of emotive language was used.	Y/PY/N/PN/NI

Suggested algorithm for rating judgment of policy recommendations level in domain 1:

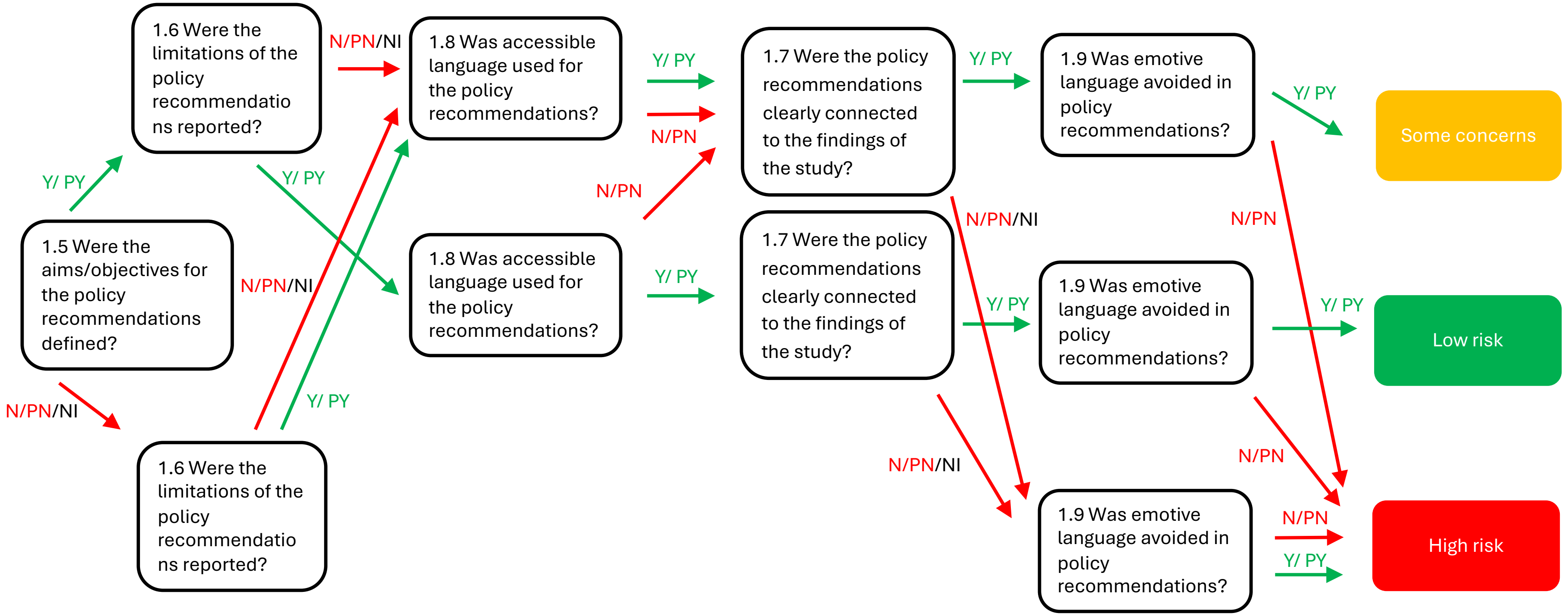

Domain 2: Offer balance, not false balance

Study level

Signalling question	Elaboration	Responses
2.1 Were all aspects of the study findings reported?	Partial representation of evidence can affect the trustworthiness of the study. Detailed and comprehensive reporting is essential for a reader to be able to make an objective interpretation of the findings. Look at text, tables and figures to determine if findings have been reported in detail.<br>Answer ‘Y/PY’ if findings are reported in both textual and tabular form as well as in figures. Reported findings should coincide with the planned analysis as reported in the methods section of the study.	Y/PY/N/PN/NI
2.2 Was an appropriate reporting guideline used for constructing the manuscript?	In many scientific disciplines, reporting guidelines are used to ensure a study’s good reporting standard. A lot of journals require a documented use of a reporting guideline as a prerequisite for accepting a manuscript for peer-review (e.g. PRISMA guideline for reporting systematic reviews, CONSORT guidelines for reporting parallel group randomised trials, CHEERS guidance for Health Economic Evaluations, COREQ criteria for reporting qualitative research. etc.).<br>Answer ‘Y/PY’ if the use of a specific reporting guideline is stated in the paper. Answer ‘NA’ in the case when, to the best knowledge of the reader, a reporting guideline for the specific scientific discipline or field does not exist.	Y/PY/N/PN/NI

Suggested algorithm for rating judgment of study level in domain 2:

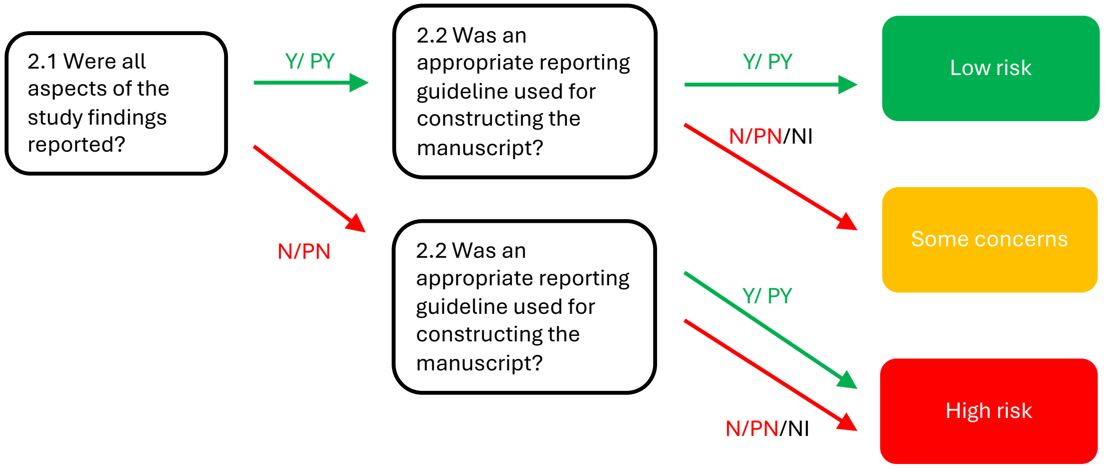

Policy recommendations level

**Signalling question**	**Elaboration**	**Responses**
2.3 Were multiple implications of the policy recommendations considered?	Policies are inherently multifactorial and can have multiple effects. A consideration of multiple outcomes of the policy recommendation shows that the researchers acknowledge the complexity of policies.<br>Moreover, the implementation of policy recommendations might have both positive and negative implications. A study should exhibit that these have been taken into consideration.<br>Answer ‘Y/PY’ if the study mentions different possible outcomes resulting from the implementation of the recommended policy/ies, ideally including pros and cons.<br>Answer ‘PY’ if the study puts forward multiple policy recommendations connected to multiple outcomes.	Y/PY/N/PN/NI
2.4 Was the existence of a current policy discussed?	In many policy areas, it might be the case that a policy is already in place for the issue the study is focusing on. Knowledge of the current policy, or of the absence of one is essential for putting forward future policy implementation.<br>Answer ‘Y’ if a current policy is identified and discussed. Answer ‘PY’ if a current policy is identified.<br>Answer ‘Y’ if the study states that a currently a policy is not in place.	Y/PY/N/PN/NI
If Y/PY to 2.4<br>2.4.1 Was not changing the current policy considered?	The outcomes of not changing this policy, which is essentially the baseline, should be considered by the study.<br>Answer ‘Y/PY’ if the effects of not changing the current policy is discussed and contrasted to the projected outcomes of the proposed policy recommendation/s. Answer ‘NA’ if the authors have stated that currently a policy is not in place.	Y/PY/N/PN/NI/NA

Suggested algorithm for rating judgment of policy recommendations level in domain 2:

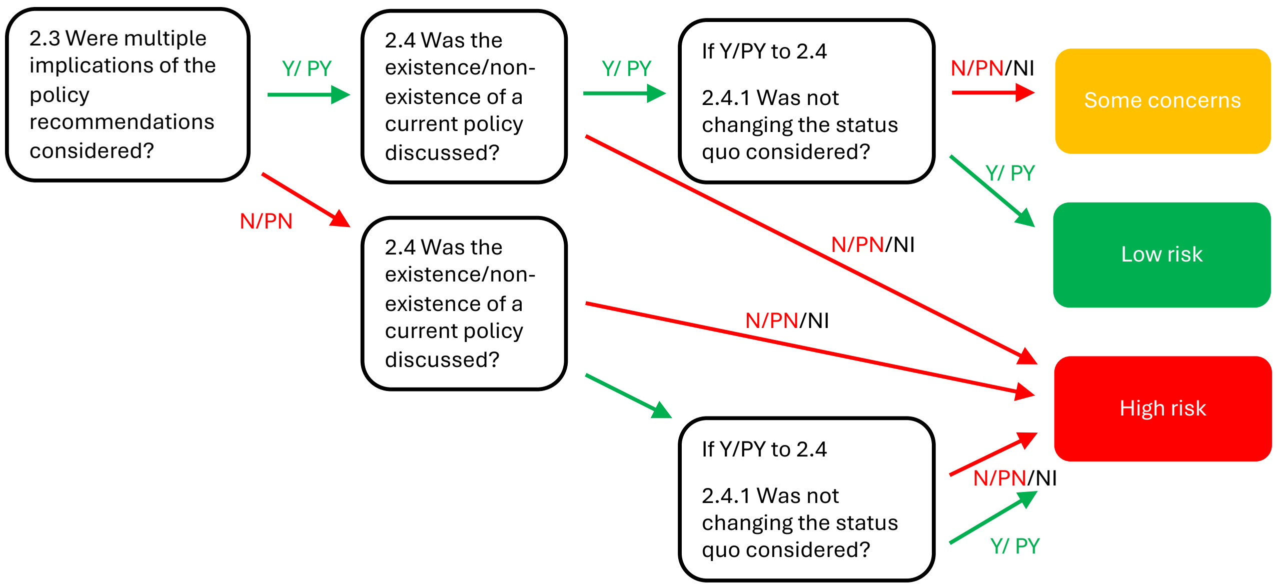

Domain 3: Disclose uncertainties

Study level

**Signalling question**	**Elaboration**	**Responses**
3.1. Were uncertainties of the study findings reported?	Disclosing uncertainties in study findings speaks to the trustworthiness of the research evidence. In simpler terms researchers should acknowledge what they don't know.<br>Answer 'Y/PY' if the study reported the uncertainties of their findings (study results).<br>Regarding numerical findings (statistical), answer 'Y/PY' if the study reported an uncertainty measurement such as standard deviation (SD), standard error (SE), confidence interval (CI), quantiles, statistical significance, or another measure of uncertainty which was appropriate for the type of outcome.<br>Uncertainty can also be presented in figures using a variety of representations e.g. error bars, shaded areas in graphs etc.<br>The measure of uncertainty should ideally be accompanied by a textual explanation of their meaning. This is particularly important when the range of the uncertainty is broad which is context dependent.<br>If the outcomes of a study are textual only, answer 'Y/PY' if the study reported uncertainty narratively.<br>A distinction should be made between the limitations and the uncertainties of study findings, addressed by signalling question 1.2 and 3.1, respectively. Limitations of study findings relate to methodology, data input and analysis and might result in uncertainties in the findings, but are distinct to them. Uncertainties relate to the difference between the research findings and the 'true values' and help us understand the degree of confidence in the study findings.	Y/PY/N/PN/NI
If Y/PY to 3.1<br>3.1.1 Did the study propose ways to reduce uncertainties in the future?	The next step after acknowledging uncertainties is proposing possible solutions in future research. Answer 'Y/PY' if the study proposes specific examples of how uncertainties might be tacked by future research.	Y/PY/N/PN/NI/NA

Suggested algorithm for rating judgment of study level in domain 3:

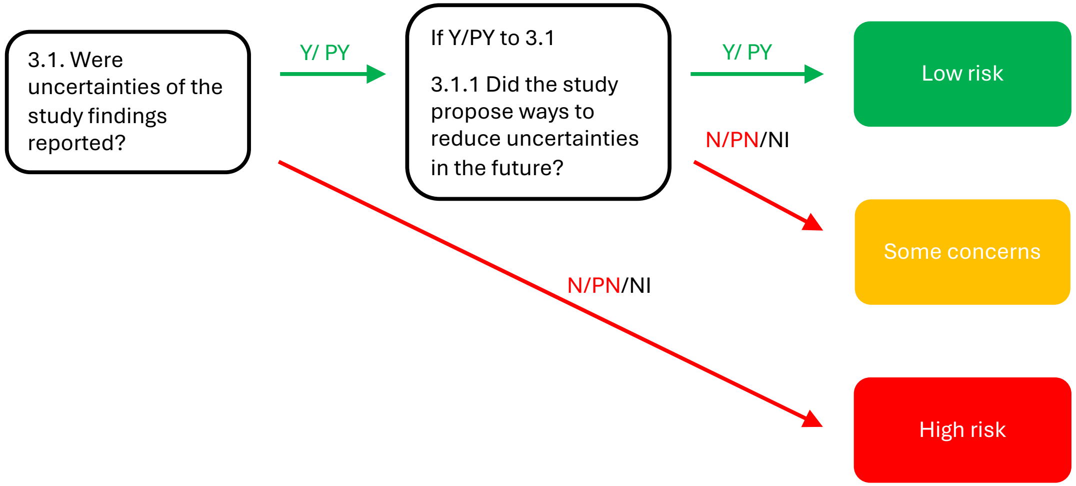

Policy recommendations level

Signalling question	Elaboration	Responses
3.2 Were uncertainties of the policy recommendations reported?	Similarly to signalling question 3.1, the study should acknowledge uncertainties in the policy recommendations. The uncertainties might be connected to the recommendations or to their outcomes and can be expressed either numerically or narrative depending on the context.<br>Answer 'Y/PY' if the study reports uncertainties in policy recommendations.	Y/PY/N/PN/NI
If Y/PY to 3.2<br>3.2.1 Did the study adopt a precautionary principle perspective?	According to the precautionary principle "When an activity raises threats of harm to human health or the environment, precautionary measures should be taken even if some cause-and-effect relationships are not fully established scientifically. The statement went on to list four central components of the principle: taking preventive action in the face of uncertainty; shifting the burden of proof to the proponents of an activity; exploring a wide range of alternatives to possibly harmful actions; and increasing public participation in decision making." [1].<br>Answer 'P/PY' if the study proposes policy recommendation in spite of uncertainties based on the precautionary principle.	Y/PY/N/PN/NI/NA

Suggested algorithm for rating judgment of policy recommendations level in domain 3:

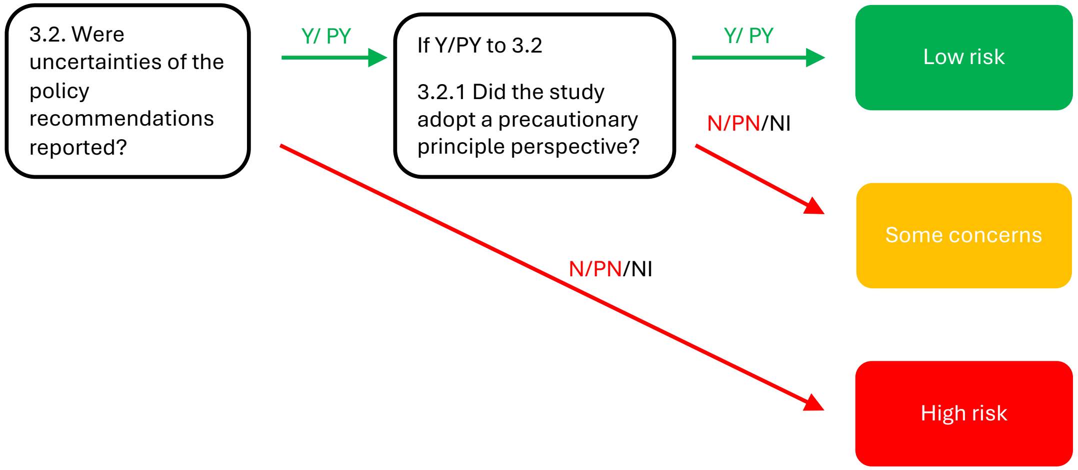

Domain 4: State evidence quality

Study level

Signalling question	Elaboration	Responses
4.1 Was the quality of the evidence used in the analysis considered?	Indirect uncertainty in a study can be caused by the underlying quality of the evidence used in the analysis. A study must consider the quality of the evidence base used in the analysis.<br>Answer ‘Y/PY’ if the study has considered the quality of the evidence base. This includes the case when a study uses data from sources that cannot be easily assessed without further investigation (e.g. data available by commercial companies) and when a study identifies the used data as the ‘only available’.<br>Answer ‘PY’ when the study reports (and appropriately references) that the evidence used in the analysis are taken from publicly available data sources distributed by organisations of acknowledged impartiality and quality, e.g. the World Development Indicators (WDI) from the World Bank database, health related data from the WHO etc. Also answer ‘PY’ if the evidence is taken from peer-reviewed papers.<br>When a mix of data source types are used, individual consideration of data quality must be present.	Y/PY/N/PN/NI
If Y/PY to 4.1<br>4.1.1 Were specific metrics of evidence quality used?	Evidence quality can be assessed using standardized metrics and tools (e.g. GRADE system for evidence of systematic reviews) or be explored in narrative manner. The use of a standardised method (if one is available for the specific scientific discipline or field) further strengthens the confidence in the evidence quality assessment outputs.<br>Answer ‘Y/PY’ if a specific metric for evidence quality has been used or if the authors explored evidence quality narratively.<br>Use ‘NI’ when the assessor is not aware of a specific available metric and there is no mention by the authors.	Y/PY/N/PN/NI/NA

Suggested algorithm for rating judgment of study level in domain 4:

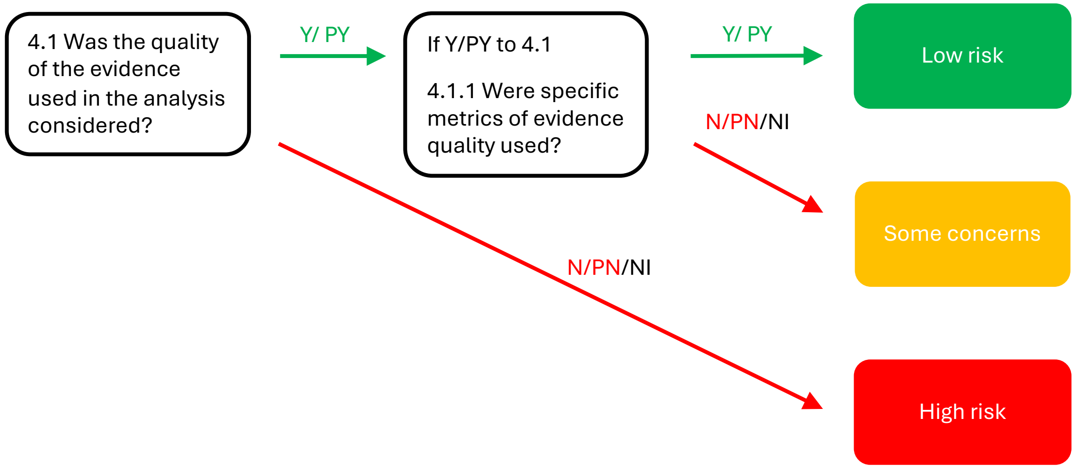

Policy recommendations level

**Signalling question**	**Elaboration**	**Responses**
4.2 Was the quality of the study findings, that formulated the evidence base for the policy recommendations, considered?	Assuming that the policy recommendations put forward by the study are connected to the study findings (also see signalling question 1.7), it is anticipated that the quality of the findings might affect the quality of the policy recommendations.<br>Answer 'Y/PY' if the quality of study findings, that formulated the evidence base for the policy recommendations was considered.<br>A 'NI' option should be used when the quality of the evidence was considered in the study level but not explicitly mentioned in the policy recommendation section as well.	Y/PY/N/PN/NI

Suggested algorithm for rating judgment of policy recommendations level in domain 4:

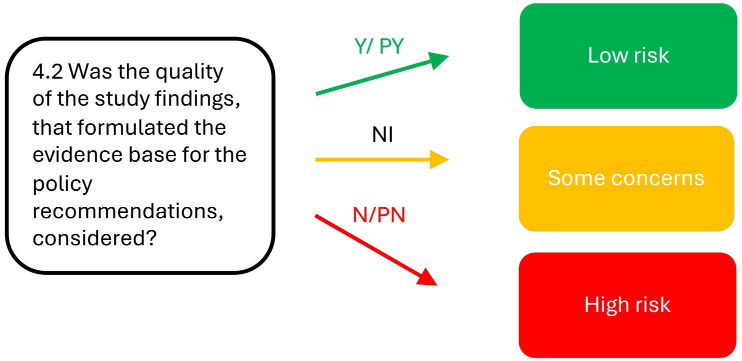

Domain 5: Pre-empting misunderstandings

Study level

**Signalling question**	**Elaboration**	**Responses**
5.1 Were potential misunderstandings about the study findings and conclusions pre-emptively addressed?	Clear and unambiguous reporting of study findings and conclusions is essential and is often the topic of critique within the peer-review process. Anticipating and pre-emptively inoculating against misunderstandings, misinformation or even disinformation is the focus of this signalling question. Transparent reporting of study limitations and uncertainties, which is championed by this critical appraisal tool, might be maliciously used by others to cast doubt. Nevertheless, this should not deter researchers from reporting them. Answer 'Y/PY' if a direct quote to this effect is found in the study. If a direct quote cannot be located in the study, look at the entire paper as a whole to make a judgement on the reporting 'style'. Is there any ambiguity in the findings and/or conclusions? If there is, answer 'N/PN'.	Y/PY/N/PN/NI

Suggested algorithm for rating judgment of study level in domain 5:

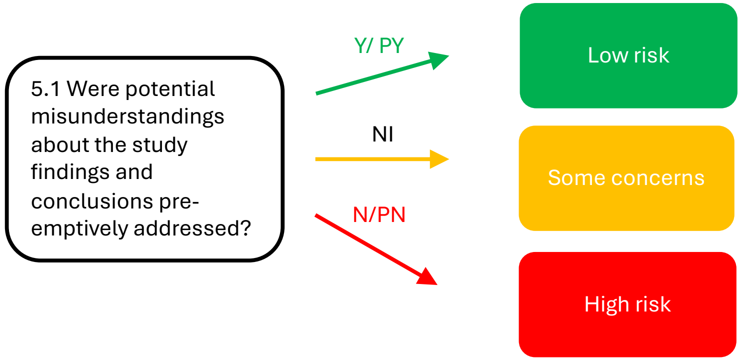

Policy recommendations level

Signalling question	Elaboration	Responses
5.2 Was the targeted audience for policy recommendations defined?	The first step in pre-emptying misunderstandings is to correctly identify the audience for the policy recommendation/s, their needs and expectations. Answer 'Y' if specific policymakers are identified. Answer 'PY' if areas of policymaking are identified.	Y/PY/N/PN/NI
5.3 Were potential misunderstandings for policy recommendations and potential concerns of the policy makers pre-emptively addressed?	Similarly to signalling question 5.1, policy recommendation/s should be clear and unambiguous, tailored to the needs of the policymakers. Phrases pre-emptively addressing potential misunderstandings and concerns might be used. Answer 'Y' if direct quotes to this effect are identified. Answer 'PY' If the policy recommendations are not vague and include specific practical examples, thus providing added clarity.	Y/PY/N/PN/NI

Suggested algorithm for rating judgment of policy recommendations level in domain 5:

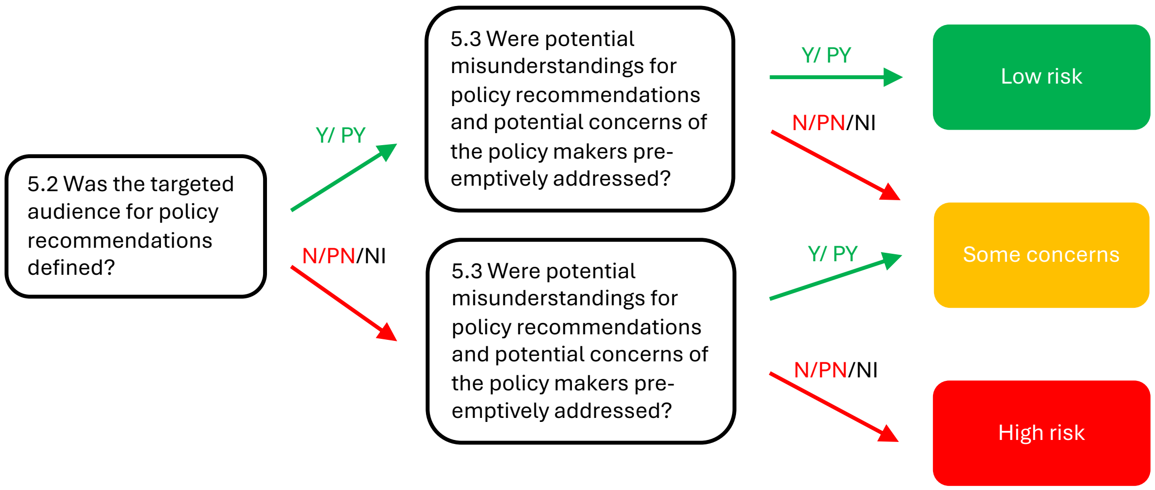

Reaching rating per domain

In combining the ratings of the two levels for each domain, the two following algorithms may be used. In domains 1, 2 and 3, we suggest that if at least one level is rated of 'high RoB' this judgment should be carried over to the domain rating. On the other hand, in domains 4 and 5, we suggest that the domain should be rated as of 'high RoB' only if both levels have been rated as of 'high RoB'. This difference reflects the size and the structure of the domains as well as the underlying origins of bias as described in the elaboration section of this document.

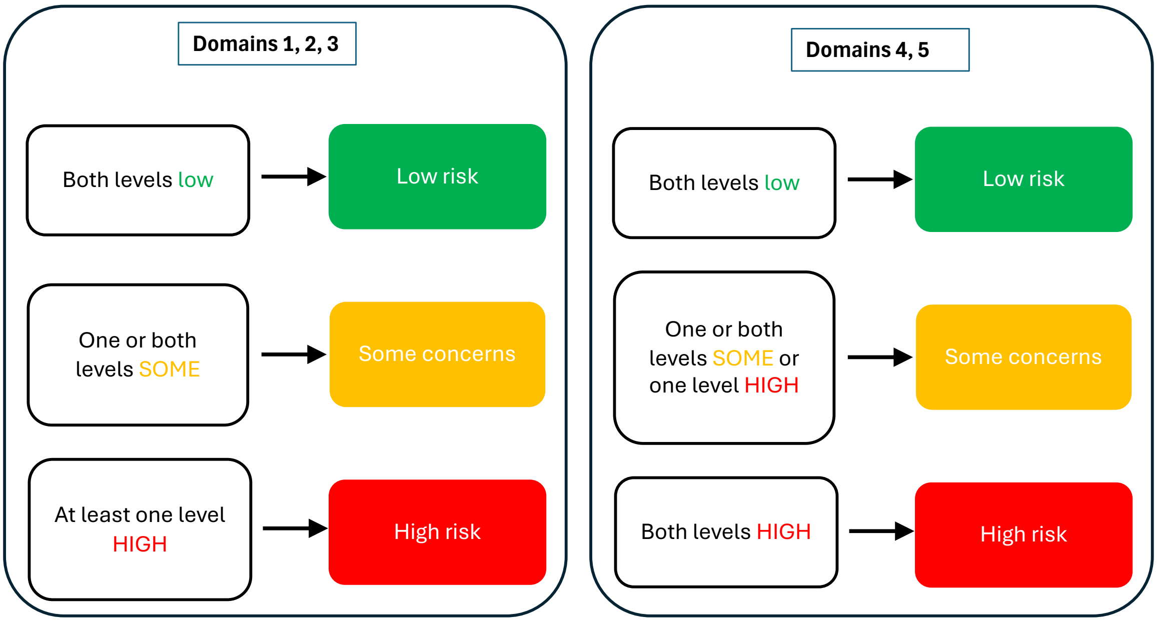

References

1. Kriebel D, Tickner J, Epstein P, Lemons J, Levins R, Loechler EL, Quinn M, Rudel R, Schettler T, Stoto M: **The precautionary principle in environmental science**. *Environ Health Perspect* 2001, 109(9):871-876.
