# Supplementary material for: Evidence Communication Rules for Policy (ECR-P) critical appraisal tool
Source: Syst Rev. 2025 Jan 13;14:10. doi: 10.1186/s13643-025-02757-8 (PMC11727712; doi:10.1186/s13643-025-02757-8)
Supplement: Supplementary file 2 — Additional file 2. ECR-P (Evidence Communication Rules for Policy) critical appraisal tool. Mapping of the ECR-P (Evidence Communication Rules for Policy) critical appraisal tool signalling questions to the three dimensions of quality: internal validity, external validity, evidence communication. Mapping to evidence communication is further specified for either element of evidence veracity or communication quality. [file 13643_2025_2757_MOESM2_ESM.pdf]

ECR-P (Evidence Communication Rules for Policy) critical appraisal tool

Additional file 2

Mapping of the ECR-P (Evidence Communication Rules for Policy) critical appraisal tool signalling questions to the three dimensions of quality: internal validity, external validity, evidence communication. Mapping to evidence communication is further specified for either element of evidence veracity or communication quality.

Domain and signalling question	Mapping to quality dimensions
**Domain 1: Inform not persuade**	
1.1 Were the aims/objectives for the study defined?	Evidence communication (CQ)
1.2 Were the limitations of the study findings reported?	Internal validity/ Evidence communication (EV)
If Y/PY to 1.2:<br>1.2.1 Did the study propose ways to reduce limitations in the future?	Internal validity/ Evidence communication (CQ)
1.3 Were the study conclusions clearly connected to the findings of the study?	Internal validity
1.4 Was emotive language avoided in communicating study findings and/or conclusions?	Evidence communication (CQ)
1.5 Were the aims/objectives for the policy recommendations defined?	Evidence communication (CQ)
1.6 Were the limitations of the policy recommendations reported?	Internal validity/ Evidence communication (EV)
1.7 Were the policy recommendations clearly connected to the findings of the study?	Internal validity
1.8 Was accessible language used for the policy recommendations?	Evidence communication (CQ)
1.9 Was emotive language avoided in policy recommendations?	Evidence communication (CQ)
**Domain 2: Offer balance, not false balance**	
2.1 Were all aspects of the study findings reported?	Internal validity
2.2 Was an appropriate reporting guideline used for constructing the manuscript?	Evidence communication (EV)
2.3 Were multiple implications of the policy recommendations considered?	Internal validity
2.4 Was the existence of a current policy discussed?	External validity

If Y/PY to 2.4<br>2.4.1 Was not changing the current policy considered?	External validity/ Evidence communication (EV)
**Domain 3: Disclose uncertainties**	
3.1. Were uncertainties of the study findings reported?	Internal validity/ Evidence communication (EV)
If Y/PY to 3.1<br>3.1.1 Did the study propose ways to reduce uncertainties in the future?	Internal validity/ Evidence communication (EV)
3.2 Were uncertainties of the policy recommendations reported?	Internal validity/ Evidence communication (EV)
If Y/PY to 3.2<br>3.2.1 Did the study adopt a precautionary principle perspective?	Internal validity/ Evidence communication (EV)
**Domain 4: State evidence quality**	
4.1 Was the quality of the evidence used in the analysis considered?	Internal validity/ Evidence communication (EV)
If Y/PY to 4.1<br>4.1.1 Were specific metrics of evidence quality used?	Internal validity/ Evidence communication (CQ)
4.2 Was the quality of the study findings, that formulated the evidence base for the policy recommendations, considered?	Internal validity/ Evidence communication (EV)
**Domain 5: Pre-empty misunderstandings**	
5.1 Were potential misunderstandings about the study findings and conclusions pre-emptively addressed?	Evidence communication (CQ)
5.2 Was the targeted audience for policy recommendations defined?	Evidence communication (CQ)
5.3 Were potential misunderstandings for policy recommendations and potential concerns of the policy makers pre-emptively addressed?	External validity/ Evidence communication (CQ)

CQ, communication quality; EV, evidence veracity

Internal validity
External validity
Evidence communication
External validity/ Evidence communication
Internal validity/ Evidence communication
